# Supplementary material for: Novel Driver Strength Index highlights important cancer genes in TCGA PanCanAtlas patients
Source: PeerJ. 2022 Aug 11;10:e13860. doi: 10.7717/peerj.13860 (PMC9375969; doi:10.7717/peerj.13860)
Supplement: Supplemental Information 2 [file peerj-10-13860-s002.zip › COHORTS/genes plots/detailed/2021_8_16_14_9_distribution_events_detailed_females_15.pdf]

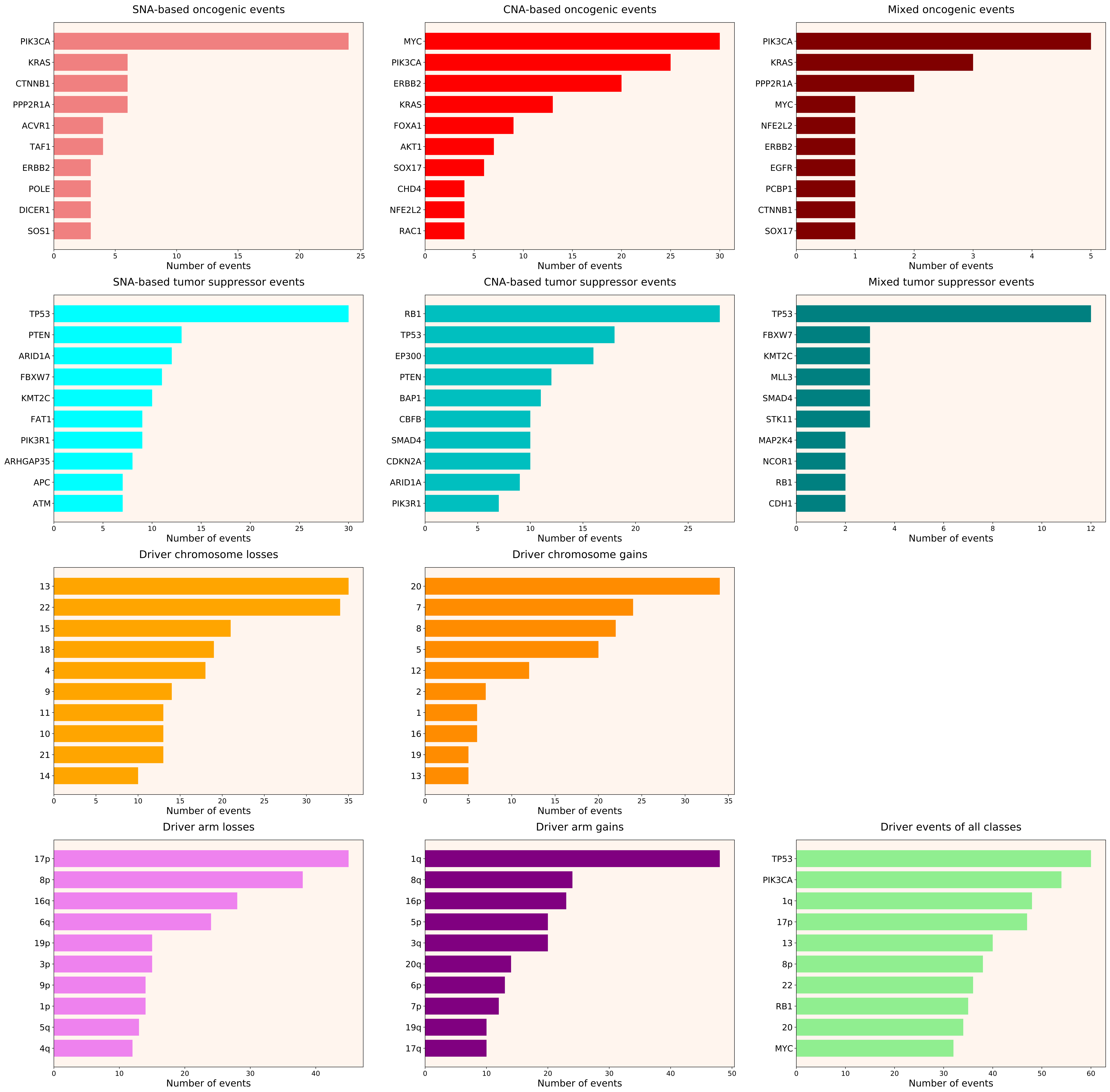
SNA-based oncogenic events
PIK3CA
KRAS
CTNNB1
PPP2R1A
ACVR1
TAF1
ERBB2
POLE
DICER1
SOS1
Number of events
CNA-based oncogenic events
MYC
PIK3CA
ERBB2
KRAS
FOXA1
AKT1
SOX17
CHD4
NFE2L2
RAC1
Number of events
Mixed oncogenic events
PIK3CA
KRAS
PPP2R1A
MYC
NFE2L2
ERBB2
EGFR
PCBP1
CTNNB1
SOX17
Number of events
SNA-based tumor suppressor events
TP53
PTEN
ARID1A
FBXW7
KMT2C
FAT1
PIK3R1
ARHGAP35
APC
ATM
Number of events
CNA-based tumor suppressor events
RB1
TP53
EP300
PTEN
BAP1
CBFB
SMAD4
CDKN2A
ARID1A
PIK3R1
Number of events
Mixed tumor suppressor events
TP53
FBXW7
KMT2C
MLL3
SMAD4
STK11
MAP2K4
NCOR1
RB1
CDH1
Number of events
Driver chromosome losses
13
22
15
18
4
9
11
10
21
14
Number of events
Driver chromosome gains
20
7
8
5
12
2
1
16
19
13
Number of events
Driver arm losses
17p
8p
16q
6q
19p
3p
9p
1p
5q
4q
Number of events
Driver arm gains
1q
8q
16p
5p
3q
20q
6p
7p
19q
17q
Number of events
Driver events of all classes
TP53
PIK3CA
1q
17p
13
8p
22
RB1
20
MYC
Number of events
